# Impact of multidisciplinary collaborative jejunal nutrition care on nutritional status and quality of life in severely burned patients

**DOI:** 10.1097/MD.0000000000041965

**Published:** 2025-04-04

**Authors:** Fang Zou, Dan Sun, Jiang Chang, Chonggen Huang, Qing Zhou, Lingtao Ding

**Affiliations:** a Burn Intensive Care Unit, The Affiliated Hospital of Jiangnan University, Jiangsu, Wuxi, China; b Department of Burns and Plastic Surgery, The Affiliated Hospital of Jiangnan University, Jiangsu, Wuxi, China.

**Keywords:** multidisciplinary collaborative based jejunal nutrition care, nutritional status, quality of life, severe burns

## Abstract

To access the impact of multidisciplinary collaborative jejunal nutrition care on the nutritional status, pain level, wound healing, and quality of life in severely burned cases. A total of 120 cases with severe burns who visited our hospital from January 2021 to May 2023 were enrolled. Inclusion criteria: ① severe burn diagnosis; ② admission within 18 hours; ③ normal heart, liver, kidney, and cognitive function; ④ informed consent signed. Exclusion criteria: ① consumptive/metabolic diseases; ② malignant tumors; ③ midway death; ④ pregnant or lactating women. Patients were divided into 2 groups: control (n = 60) receiving parenteral nutrition and observation (n = 60) receiving multidisciplinary collaborative jejunal nutrition care. The latter included a multidisciplinary team (burns, endocrinology, cardiology, nephrology) and professionally trained caregivers. The jejunal nutrition care included the placement of a nasojejunal tube, individualized nutrition infusion protocols, close monitoring of vital signs, psychological counseling, and regular consultations with the multidisciplinary team. Outcomes assessed included hemoglobin, transferrin, albumin levels, pain (Visual Analogue Scale), wound healing, complications, SF-36 quality of life, and nursing satisfaction. After management, the observation group indicated significantly higher levels of hemoglobin (mean difference: 16.28 g/L, 95% CI: 12.5–20.1; Cohen d: 2.0, 95% CI: 1.7–2.3), transferrin (mean difference: 0.67 g/L, 95% CI: 0.5–0.9; Cohen d: 2.5, 95% CI: 2.1–3.0), and albumin (mean difference: 5.26 g/L, 95% CI: 4.2–6.3; Cohen d: 3.5, 95% CI: 3.0–4.0) compared to the control group (*P* < .05). The Visual Analogue Scale scores in the observation group were significantly lower (mean difference: 3.18 points, 95% CI: 2.8–3.5; Cohen d: 6.5, 95% CI: 5.9–7.2), and the wound healing time was significantly shorter (mean difference: 7.41 days, 95% CI: 4.5–10.3; Cohen d: 0.8, 95% CI: 0.6–1.0, *P* < .05). The observation group showed a lower complication rate (*P* = .02). Additionally, the observation group demonstrated significant improvements in SF-36 quality of life scores and higher nursing satisfaction (96.67% vs 80.00%, *P* = .0001). Multidisciplinary collaborative jejunal nutrition care effectively improves nutritional status, reduces pain, accelerates wound healing, and enhances quality of life and satisfaction in severely burned patients.

## 1. Introduction

Burns are a common and often severe clinical condition, primarily caused by scalds from boiling water and fire-related injuries. They frequently result in complications such as wound expansion, infection, and permanent scarring.^[1]^ Beyond direct tissue damage, burns induce physiological stress, disrupting the skin barrier and increasing the potential for bacterial colonization, which complicates recovery. The impaired immune function in burn patients makes them particularly vulnerable to infections from pathogenic bacteria, which can lead to sepsis, a life-threatening condition.^[2]^ Severe burns are typically defined as burns affecting ≥ 30% of the total body surface area or third-degree burns covering 10% to 19% of the total body surface area, or a combination of second- and third-degree burns with complications such as aspiration pneumonia, systemic shock, or multiple injuries.^[3]^

The treatment of severe burns is challenging due to these complications. Severe burn injuries place patients in a state of high metabolic stress, characterized by increased catabolism and decreased anabolism, leading to significantly higher energy demands. If not adequately addressed, this imbalance can impede wound healing and increase the risk of further complications.^[4]^ Proper nutritional support is essential for burn recovery, providing the necessary caloric intake, promoting tissue repair, supporting immune function, and improving overall prognosis.^[5]^ While traditional parenteral nutrition (PN) is commonly used to meet these needs, prolonged PN can lead to adverse effects such as gastrointestinal mucosal atrophy and a higher risk of infections, including pneumonia and gastrointestinal bleeding. These risks limit the long-term effectiveness of PN and highlight the need for alternative nutritional strategies.^[6,7]^

Enteral nutrition (EN), particularly jejunal nutrition, offers a more physiological approach to nutritional support. Unlike gastric feeding, jejunal nutrition bypasses the stomach, reducing the risks of aspiration and gastric reflux, which are common complications in burn patients. Moreover, jejunal feeding enhances gut microcirculation, lowers the likelihood of upper gastrointestinal bleeding, and helps maintain intestinal function, making it a more suitable option for severely burned individuals.^[8]^ However, jejunal nutrition requires careful management due to the challenges posed by burn patients’ conditions, including fluid loss, pain, and infection.

A promising solution to these challenges is the multidisciplinary cooperative nursing model, which has been successfully applied in managing various diseases. This model promotes collaboration among specialists from multiple medical fields, ensuring comprehensive care tailored to the patient’s needs. In this study, the multidisciplinary team includes experts in burn care, endocrinology, cardiology, and nephrology, working alongside trained caregivers. The goal is to provide holistic care that addresses both the nutritional requirements and the complex medical issues faced by burn patients. This model also incorporates psychological support to improve patient cooperation and compliance, both of which are essential for achieving successful treatment outcomes.^[9]^ By integrating medical, nutritional, and psychological care, the model enhances recovery and contributes to better overall patient outcomes.

Currently, the standard nutritional support for burn patients typically involves a combination of parenteral and enteral nutrition, with the choice based on the patient’s clinical condition. Factors such as gastrointestinal dysfunction, aspiration risk, and the patient’s tolerance to different feeding methods determine the decision between parenteral and enteral nutrition. Jejunal nutrition, investigated in this study, offers an alternative to traditional gastric feeding, which may be less effective for burn patients with compromised gastric motility or a higher risk of aspiration.^[8,9]^

This purpose of this research is to assess the effectiveness of multidisciplinary collaborative jejunal nutrition care in improving nutritional status, pain management, wound healing, and quality of life in severely burned patients. We hypothesize that this approach will significantly outperform conventional PN in clinical outcomes and patient satisfaction, providing a valuable alternative for managing severe burns in clinical practice.

## 2. Material and methods

### 2.1. General material

This research was approved by the Ethics Committee of The Affiliated Hospital of Jiangnan University on January 8, 2024 (Ethics No. 2024 No. 27). All 120 severe burn patients who were admitted to our hospital from January 2021 to May 2023 were selected as the research subjects. These patients were randomly assigned to 2 groups: the control group (60 cases) and the observation group (60 cases). Random allocation was performed using a computer-generated random number method, where each patient was assigned a unique number, and the allocation to either the control or observation group was determined by the random number sequence, ensuring unbiased distribution between the groups. The control group received PN support, while the observation group received jejunal nutrition support. There was no statistical difference between the 2 groups in terms of sex, age, burn area, time from burn to treatment, and cause of burn (*P* > .05), confirming that the groups were comparable at baseline, as shown in Table 1.

**Table 1:** Comparison of 2 groups of general material.

General material		Control group (n = 60)	Observation group (n = 60)	*t*/*χ*^2^ value	*P*-value
Gender [n (%)}	Male	34 (56.67)	26 (60.00)	0.023	.887
	Female	26 (43.33)	24 (40.00)		
Average age (years)		45.15 ± 9.17	45.23 ± 9.28	0.372	.708
Average time from burn to medical treatment (h)		5.35 ± 2.05	5.33 ± 1.96	0.459	.639
Average burn area (%)		47.35 ± 10.06	47.45 ± 10.08	1.099	.275
Cause of burn [n (%)]	Flame burn	26 (43.33)	30 (50.00)	0.473	0.492
	Hydrothermal burn	20 (33.33)	18 (30.00)		
	Chemical burn	14 (23.33)	12 (20.00)		

### 2.2. Criteria of inclusion and exclusion

Inclusion criteria: ① all patients were diagnosed with severe burns based on the China 9-point method, palm method, and other assessment methods; ② patients admitted to the hospital within 18 hours after sustaining burns; ③ cases with normal important organ functions; ④ patients with normal cognitive function, as assessed by the Mini-Mental State Examination or a similar cognitive assessment tool, with a score within the normal range for their age group. Patients with normal spirit, by which we mean patients who are alert and oriented, without signs of severe psychological distress or major psychiatric disorders that would interfere with the study; ⑤ patients or family members gave informed consent for the study.

Exclusion criteria: ① cases with consumptive or metabolic diseases; ② patients with malignant tumors; ③ patients who died before completing the full treatment protocol (i.e., before receiving the entirety of the study interventions); ④ pregnant or lactating women. *Note:* No cases in either the control or observation group died during the course of the study.

### 2.3. Methods

First, patients in both groups received the standard treatment for severe burns. Patients were stabilized and underwent acute-phase treatment, including initial resuscitation, wound management, and monitoring of vital signs, before being included in the study. This stabilization period typically occurred within 48 to 72 hours after admission, ensuring that patients were in a stable condition (e.g., blood pressure, heart rate, respiratory function, and fluid balance were maintained within normal ranges).

The control group was given PN support nursing for 3 days: firstly, the venous passage was established, and the appropriate amount of electrolyte, glucose, fat emulsion, vitamins, and amino acids were pumped into the patient’s body from deep vein or vein at a uniform speed through a micropump. When the patient’s gastrointestinal tract became intolerant, the dripping speed was slowed down and the total amount of pumping was reduced, so that the patient could take intestinal Bifidobacterium (Jincheng Hayes Pharmaceutical Co., Ltd., National Medicine Zhunzi S19993065) or motilium (Xi’an Janssen Pharmaceutical Companies Co., Ltd., National Medicine Zhunzi). In addition, psychological counseling and vital signs monitoring were given. This nutritional support was provided for 3 days, after which the patient was monitored for any signs of tolerance before any adjustments were made to the treatment.

The observation group was given multidisciplinary collaborative based jejunal nutrition care, set up a multidisciplinary cooperative group, including a physician in the burn department, endocrinology department, cardiology department, and nephrology department, who can consult at any time. Also includes a head nurse, several professionally trained caregivers. The nursing intervention protocol was developed jointly by a multidisciplinary collaborative group, as follows:

① Ultrasound-guided nasojejunal tube insertion was performed. Model of nasojejunal tube: Folkai spiral nasojejunal tube, CH10-145, produced by Newdixia Pharmaceutical Co., Ltd. The nasojejunal nutrition tube was disposed 5 to 10 cm distal to the Treitz ligament in the descending segment of duodenum, and then contrast agent was injected into the gastric tube to understand the position of the nutrition tube under fluoroscopy. When it was confirmed that the patient did not suffer from aggravation of abdominal pain and distension, 500 mL of propanil (Nutricia Pharmaceutical (Wuxi) Co., Ltd.) was infused on the first day. National Drug Administration approval No. H20010285). Meanwhile, the infusion pump is used to control the infusion speed. The initial infusion speed is controlled at about 25 mL/h, and then it can be doubled every 24 hours until it increases to 100 mL/h. Then, according to the specific tolerance of patients, the dose of beprid was adjusted to 1000 to 1500 mL. The results of computed tomography examination of the abdomen suggested that the exudates around the pancreas were significantly reduced, and the results of blood amylase reexamination showed normal. The catheter could be removed 2 to 3 days later, and the patient could gradually transition to a normal diet. ② Communicate with patients and family members well before catheterization, explain the purpose, necessity and expected effect of jejunum nutrition, and explain the role of jejunum nutrition tube to them at the same time, in order to reduce their rejection and improve compliance. The process, method, and precautions of jejunal catheterization can be explained to the patient through the combination of plain language and pictures, and psychological counseling and encouragement can be given to increase their confidence in treatment. During the implementation of enteral nutrition, attention should be paid to control the infusion flow rate and frequency of the nutrient solution, and the length of the nutrient tube should be recorded daily to judge the position of the nutrient tube in the digestive tract in time. Pay attention to the nutrition tube fixed, in order to avoid its fall off, distortion, shift, and ensure the pipe unobstructed. In the process of nutrient solution infusion, the height of the patient’s head could be taken to 30, to avoid reflux aspiration. After each nutrient solution infusion, the pipeline was rinsed with warm boiled water to avoid blockage of the nutrient tube. During the implementation of enteral nutrition, attention was paid to the oral care of patients, and coagulation function, routine blood test and electroencephalogram were checked regularly. Close attention was paid to vital signs, and psychological care was performed to meet the physical and mental needs of patients as much as possible.

### 2.4. Observational index

① Nutritional status: 6 mL of fasting elbow venous blood was collected from patients in both groups in the early morning before and 1 week after nursing. The blood was placed into EP tubes and analyzed using an automatic biochemical analyzer (Beckman Coulter, model: AU5800) to detect the levels of hemoglobin (Hb), transferrin (TRF), and albumin (ALB). ② Pain severity, wound healing time, and complication incidence: The Visual Analogue Scale (VAS)^[10]^ was used to assess pain severity in patients in both groups before and 1 week after nursing. The total score is 10, with a higher score indicating more severe pain. The incidence of complications such as stress ulcers, catheter sepsis, and wound sepsis was recorded. Assessments were made at 1 week after nursing. ③ Quality of life: the MOS Short Form Health Survey (SF-36)^[11]^ was used to assess quality of life in patients in both groups before and 4 weeks after nursing, covering 8 dimensions: mental health, emotional function, social function, vitality, overall health, bodily pain, physical function, and role functioning. Each dimension has a maximum score of 100, with a higher score indicating better quality of life. ④ Nursing satisfaction: a self-designed scale was used to assess nursing satisfaction in both groups, categorized as satisfied, relatively satisfied, and dissatisfied, with total satisfaction defined as the sum of satisfied and relatively satisfied responses. The assessment was conducted 1 week after nursing.

### 2.5. Statistical methods

Prior to participant recruitment, a sample size calculation was performed based on the primary outcome of nutritional status. The calculation was based on an expected effect size of 0.5 (representing a medium effect size), a statistical power of 80% (β = 0.2), and a significance level of 0.05 (α = 0.05). Using G*Power version 3.1, it was determined that a sample size of 60 participants per group (120 total) would be required to ensure adequate statistical power to detect meaningful differences in the primary outcomes. This information was considered when determining the target sample size for the study.

Statistical analysis was performed using SPSS 18.0. The measurement data were expressed as mean ± standard deviation (x̄ ±s). The normality of the data was assessed using the Kolmogorov–Smirnov test. Data that followed a normal distribution were analyzed using the t; enumeration data were expressed as example (N) or percentage (%) and *χ*^2^ test was conducted. *P* < .05 indicated that the difference had statistical significance.

For significant outcomes, effect sizes (Cohen d) were calculated to assess the magnitude of the differences between the groups. Cohen d values were interpreted according to standard guidelines: values of 0.2, 0.5, and 0.8 were considered to represent small, medium, and large effects, respectively. Confidence intervals for effect sizes were also reported to provide a range of plausible values for the observed effects. These confidence intervals offer further insight into the precision of the effect size estimates and help assess the stability and reliability of the observed differences.

## 3. Results

### 3.1. Comparison of nutritional status between groups before and after nursing

Before nursing, there was no significant difference in levels of Hb, TRF, and ALB between the 2 groups (*P* > .05). After nursing, levels of Hb, TRF, and ALB in both groups significantly increased. The observation group showed significantly higher levels of Hb, TRF, and ALB compared to the control group, with differences that were statistically significant (*P* < .05). The mean differences ranged from 0.67 to 16.28, and Cohen d values ranged from 2.0 to 3.5, indicating very large effect sizes for the observation group. These results suggest that the observation group experienced a substantially greater improvement in nutritional status, as shown in Table 2 and Figure 1.

**Table 2:** Comparison of nutritional status between 2 groups before and after nursing (±s, g/L).

Index		Control group (n = 60)	Observation group (n = 60)	Mean difference (95% CI)	Cohen d (95% CI)	*P*-value
Hb	Before nursing	82.13 ± 12.16	82.54 ± 12.11	0.41 (-1.0 to 1.8)	0.03 (-0.1 to 0.2)	.82
	After nursing	101.35 ± 7.13*	117.63 ± 10.46*#	16.28 (12.5 to 20.1)	2.0 (1.7 to 2.3)	.0001
TRF	Before nursing	1.77 ± 0.26	1.89 ± 0.24	0.12 (-0.1 to 0.3)	0.05 (-0.05 to 0.15)	.14
	After nursing	2.41 ± 0.33*	3.08 ± 0.45*#	0.67 (0.5 to 0.9)	2.5 (2.1 to 3.0)	.0002
ALB	Before nursing	27.11 ± 2.15	27.33 ± 2.07	0.22 (-0.3 to 0.8)	0.1 (-0.1 to 0.2)	.71
	After nursing	30.21 ± 1.52*	35.47 ± 2.24*#	5.26 (4.2 to 6.3)	3.5 (3.0 to 4.0)	.0001

ALB = albumin, Hb = hemoglobin, TRF = transferrin.

* *P* < .05, compared with that before nursing.

# *P* < .05, compared with the control group.

**Figure 1.**
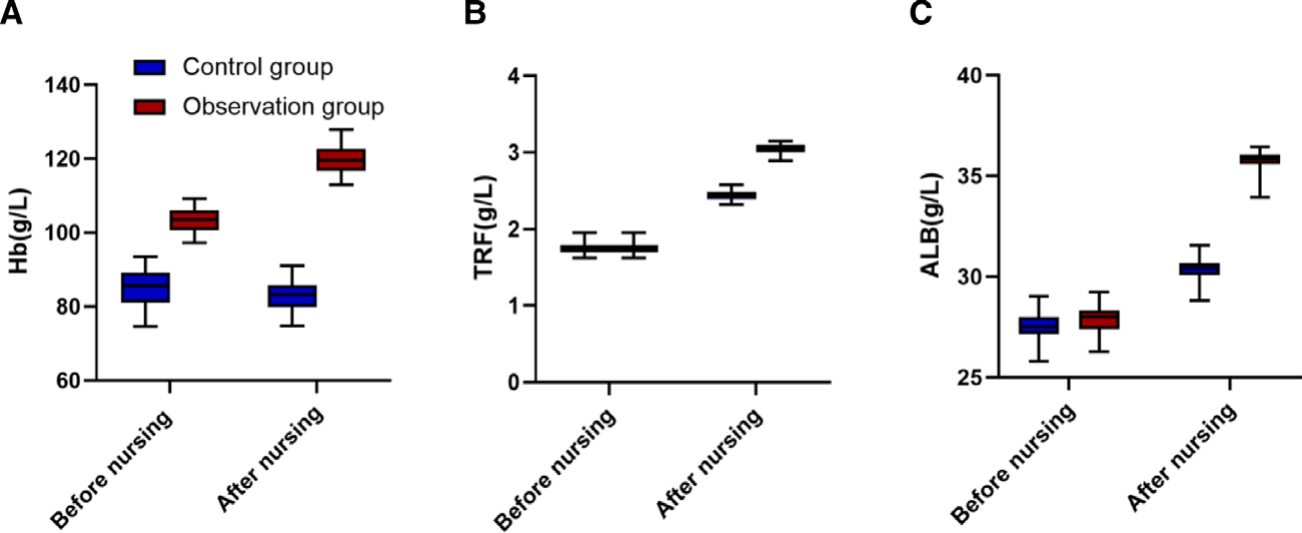
Comparison of nutritional status between 2 groups before and after nursing.

### 3.2. Comparison of pain severity, wound healing time, and incidence of complications between groups

After nursing, the VAS scores of both groups were significantly reduced, with the observation group showing a significantly lower score (*P* < .05). Specifically, the mean difference in VAS scores after nursing was 3.18 points (95% CI: 2.8–3.5), and the Cohen d was 6.5 (95% CI: 5.9–7.2), indicating a large effect size.

The time of wound healing in the observation group was significantly shorter, with a mean difference of 7.41 days (95% CI: 4.5–10.3) and Cohen d of 0.8 (95% CI: 0.6–1.0). The incidence of complications was also significantly lower in the observation group (23.33% vs 50.00% in the control group), with a mean difference of 26.67% (95% CI: 12.9–40.4) and Cohen d of 0.75 (95% CI: 0.5–1.0). These differences were statistically significant (*P* < .05), as shown in Table 3 and Figure 2.

**Table 3:** Comparison of pain severity, wound healing time, and incidence of complications between 2 groups.

Index		Control group (n = 60)	Observation group (n = 60)	Mean difference (95% CI)	Cohen d (95% CI)	*P*-value
VAS score (points)	Before nursing	8.16 ± 0.47	8.18 ± 0.48	0.02 (-0.3 to 0.3)	0.04 (-0.2 to 0.3)	0.87
	After nursing	5.39 ± 0.39*	2.21 ± 0.42*#	3.18 (2.8 to 3.5)	6.5 (5.9 to 7.2)	0.0001
Wound healing time (d)		61.62 ± 9.44	54.21 ± 7.18*	7.41 (4.5 to 10.3)	0.8 (0.6 to 1.0)	0.0003
Incidence of complications [n (%)]		15 (50.00)	7 (23.33)*	26.67 (12.9 to 40.4)	0.75 (0.5 to 1.0)	0.02

* *P* < .05, compared with that before nursing.

# *P* < .05, compared with the control group.

**Figure 2.**
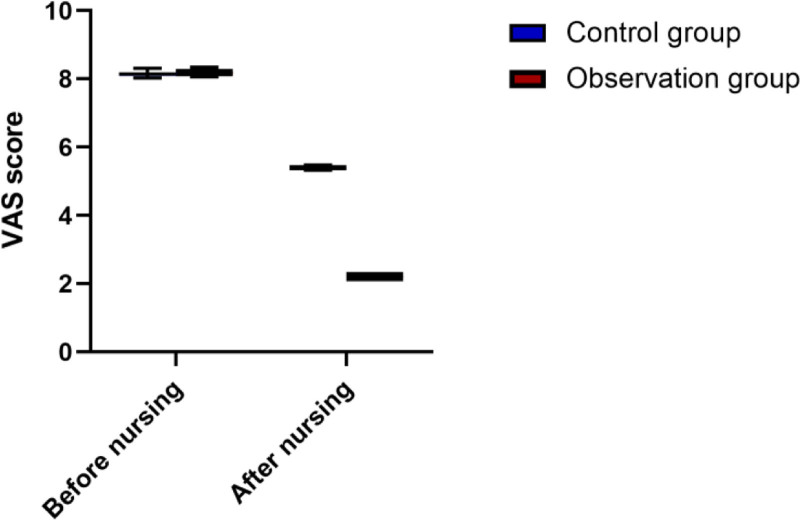
Comparison of pain levels between 2 groups before and after nursing.

### 3.3. Comparison of quality of life between groups before and after nursing

 After nursing, all SF-36 scores of both groups significantly increased, with each score in the observation group significantly higher (*P* < .05). Specifically, the largest effect sizes were observed for the following dimensions: mental health: the mean difference in the mental health score after nursing was 8.58 points (95% CI: 7.0–10.1) with a Cohen d of 1.2 (95% CI: 1.0–1.4), indicating a large effect size. Emotional function: the mean difference was 7.60 points (95% CI: 6.2–9.0) with Cohen d of 1.1 (95% CI: 0.9–1.3). Social function: the mean difference was 7.32 points (95% CI: 5.8–8.8), with Cohen d of 1.0 (95% CI: 0.8–1.2). Vitality: the mean difference was 7.94 points (95% CI: 6.3–9.6) with Cohen d of 1.2 (95% CI: 1.0–1.4). Bodily pain: the mean difference was 8.00 points (95% CI: 6.3–9.7) with Cohen d of 1.1 (95% CI: 0.9–1.3), as shown in Table 4 and Figure 3.

**Table 4:** Comparison of quality of life between 2 groups before and after nursing (±s, score).

Item		Control group (n = 60)	Observation group (n = 60)	Mean difference (95% CI)	Cohen d (95% CI)	*P*-value
Mental health	Before nursing	57.51 ± 6.23	57.21 ± 6.33	0.3 (-0.5 to 1.1)	0.05 (-0.2 to 0.3)	.76
	After nursing	78.21 ± 6.30*	86.79 ± 6.23*#	8.58 (7.0 to 10.1)	1.2 (1.0 to 1.4)	.0001
Emotional function	Before nursing	57.69 ± 6.10	57.27 ± 6.20	0.42 (-0.4 to 1.2)	0.07 (-0.3 to 0.4)	.83
	After nursing	78.99 ± 6.20*	86.59 ± 6.50*#	7.60 (6.2 to 9.0)	1.1 (0.9 to 1.3)	.0001
Social function	Before nursing	58.69 ± 6.08	58.19 ± 6.40	0.5 (-0.2 to 1.2)	0.08 (-0.3 to 0.3)	.76
	After nursing	79.89 ± 6.49*	87.21 ± 6.49*#	7.32 (5.8 to 8.8)	1.0 (0.8 to 1.2)	.0001
Vitality	Before nursing	58.50 ± 6.13	58.18 ± 6.16	0.32 (-0.4 to 1.0)	0.05 (-0.3 to 0.3)	.85
	After nursing	79.63 ± 6.06*	87.57 ± 6.49*#	7.94 (6.3 to 9.6)	1.2 (1.0 to 1.4)	.0001
Overall health	Before nursing	57.99 ± 6.21	57.21 ± 6.18	0.78 (-0.2 to 1.8)	0.12 (-0.3 to 0.5)	.61
	After nursing	78.30 ± 6.29*	86.46 ± 6.08*#	8.16 (6.6 to 9.7)	1.1 (0.9 to 1.3)	.0001
Bodily pain	Before nursing	57.20 ± 6.26	57.21 ± 6.79	-0.01 (-0.8 to 0.7)	0.00 (-0.3 to 0.3)	.99
	After nursing	78.21 ± 6.16*	86.99 ± 6.28*#	8.00 (6.3 to 9.7)	1.1 (0.9 to 1.3)	.0001
Physical role	Before nursing	57.69 ± 6.49	57.18 ± 6.23	0.51 (-0.3 to 1.3)	0.08 (-0.3 to 0.4)	.78
	After nursing	78.67 ± 6.27*	86.59 ± 6.29*#	7.92 (6.4 to 9.4)	1.1 (0.9 to 1.3)	.0001
Physical function	Before nursing	57.59 ± 6.33	57.69 ± 6.07	-0.1 (-0.8 to 0.6)	-0.02 (-0.3 to 0.2)	.84
	After nursing	78.69 ± 6.30*	86.63 ± 6.29*#	8.10 (6.5 to 9.7)	1.2 (1.0 to 1.4)	.0001

* *P* < .05, compared with that before nursing.

# *P* < .05, compared with the control group.

**Figure 3.**
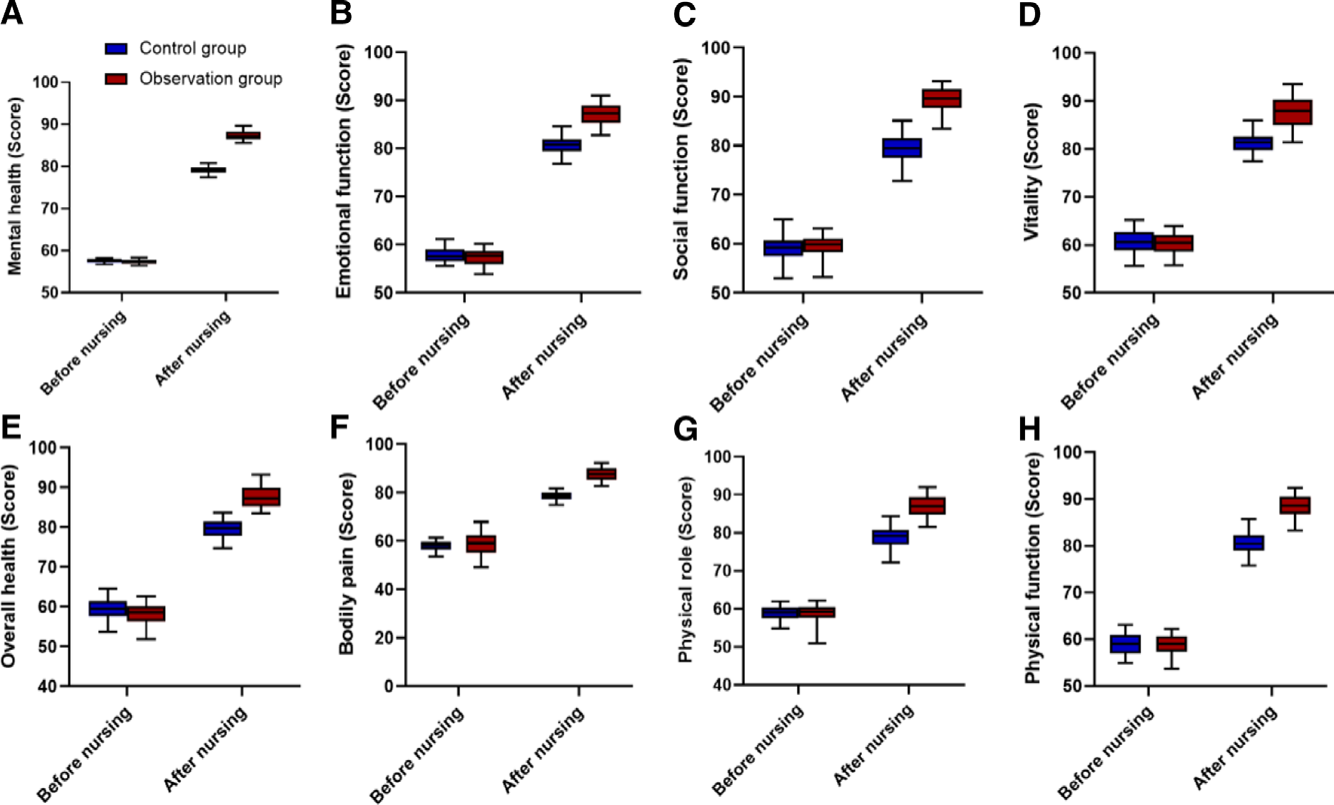
Comparison of quality of life between 2 groups before and after nursing.

### 3.4. Comparison of nursing satisfaction between groups

The degree of satisfaction with nursing in the observation group was significantly higher and the difference was statistically significant (*P* < .05), as shown in Table 5.

**Table 5:** Comparison of nursing satisfaction between 2 groups [n (%)].

Satisfaction degree	Control group (n = 60)	Observation group (n = 60)	*χ*^2^ value	*P*-value
Satisfied	28 (46.67)	36 (60.00)	–	–
Relatively satisfied	20 (33.33)	22 (36.67)	–	–
Dissatisfied	12 (20.00)	2 (3.33)	–	–
Total satisfaction	48 (80.00)	58 (96.67)	6.223	0.000

## 4. Discussion

Burns are common severe injuries in clinical practice, typically caused by high-temperature sources such as flames, hot liquids, and high-temperature machinery. Burns can lead to varying degrees of skin and deep tissue damage, triggering complex pathophysiological responses, including the release of catecholamines and 5-hydroxytryptamine, which increase capillary permeability and cause microcirculatory disturbances.^[12]^ In the case of severe burns, systemic complications such as shock, coma, respiratory distress, and multiple organ failure, including damage to the kidneys, brain, and lungs, may occur and require prompt treatment.^[13]^ Nutritional support is crucial in the recovery process of burn patients because burns lead to increased metabolic demands, fluid loss, and protein–energy malnutrition. Without timely nutritional intervention, wound healing may be delayed, the risk of infection increased, and even life-threatening complications may arise.^[14]^

Enteral nutrition (EN), particularly jejunal nutrition, is a commonly used nutritional support method, especially for patients with impaired gastrointestinal function, as it directly provides nutrients and promotes gut health.^[15–17]^ However, the physical and psychological challenges faced by burn patients often affect their nutritional intake and overall recovery. Despite this, research on the role of multidisciplinary collaborative care in combining nutritional support, clinical monitoring, and psychological care for burn patients remains limited. This study aims to fill this gap, and the results show that multidisciplinary collaborative jejunal nutrition care significantly improved the nutritional status, quality of life, pain relief, and wound healing in burn patients.

The mechanisms of improvement observed can be explained through several pathways. Firstly, enteral nutrition, by directly contacting the intestinal mucosa, promotes nutrient absorption, thereby enhancing the integrity and function of the gut.^[18]^ This not only supports better nutrient absorption but also helps maintain the gut microbiota balance, which is essential for immune function and overall recovery.^[19]^ Furthermore, jejunal nutrition stimulates the secretion of digestive enzymes and gastrointestinal hormones, accelerates the recovery of gut motility, and reduces postoperative complications such as bloating and constipation.^[18]^ In contrast, PN, while effective in providing nutritional support when gastrointestinal function is impaired, may exacerbate inflammation, weaken immune function, and increase the risk of intestinal atrophy, leading to long-term complications.^[20]^

Additionally, the improvements observed in biochemical nutrition indicators (such as Hb, TRF, ALB) and clinical outcomes (such as wound healing time and incidence of complications) indicate that the effects of the intervention are not solely attributed to the benefits of jejunal nutrition, but also to the comprehensive multidisciplinary care approach. The multidisciplinary care model combined nutritional intervention, enhanced clinical monitoring, and psychological care, all of which contributed to the rehabilitation process of burn patients. Close monitoring ensured early detection of complications, while the involvement of a multidisciplinary team (such as nutritionists and psychologists) addressed both the physical and psychological needs of the patients, thereby improving clinical outcomes and emotional well-being.^[14,21–26]^

The results align with previous research, highlighting the application of enteral nutrition in burn cases. For example, Lou et al^[16]^ found that compared with nonenteral immunonutrition support, burn patients treated with enteral immunonutrition had significantly improved TRF and ALB levels, a shorter hospitalization period, and improved patient satisfaction. Wang et al^[17]^ demonstrated that a structured nutritional care management model effectively improved ALB and TRF levels, reduced mechanical ventilation duration, and decreased the incidence of complications in severe acute pancreatitis patients receiving nasojejunal nutrition. These findings are consistent with our study, indicating the significant role of enteral nutrition in improving nutritional status and clinical outcomes. Furthermore, the results of this study are in line with those of Castanon et al,^[27]^ who found that early enteral nutrition could improve the prognosis of elderly burn patients by enhancing immune function and reducing the incidence of infections.

In this study, the multidisciplinary collaborative care model, in addition to providing enteral nutrition, included close clinical monitoring and psychological support, which may further explain the favorable recovery outcomes in the observation group. This integrated care model not only focuses on nutritional support but also involves wound care, psychological counseling, and regular patient follow-up, helping to improve recovery from multiple aspects. This integrated care model has also been validated in other clinical studies, which have shown that collaborative team care can improve patient satisfaction, reduce complications, and accelerate recovery.^[28,29]^

From a pragmatic perspective, it is important to consider the resource-intensive nature of the interventions used in this study. The multidisciplinary collaborative model requires the participation of multiple professionals and close monitoring of the patient’s condition, which undoubtedly increases the consumption of medical resources and may limit its widespread application in resource-constrained regions or healthcare environments. The demand for specialists and equipment raises the question of whether such interventions are feasible for large-scale application.

Nevertheless, in hospitals with sufficient medical resources, implementing this customized care intervention model still holds potential. For example, in high-income countries or hospitals with advanced burn treatment facilities, a trained team of nutritionists, psychologists, and burn specialists may provide a more cost-effective model while still significantly improving patient clinical outcomes. Future studies should explore the cost-effectiveness and scalability of such interventions to determine whether they can be applied in various clinical settings.

The limitations of this research include the small sample size, which may limit the generalizability of the findings. Additionally, the lack of long-term follow-up prevents the determination of the long-term effects of multidisciplinary collaborative jejunal nutrition care on burn patients’ recovery and quality of life. Future research should expand the sample size, conduct multicenter studies, and incorporate long-term follow-up to better assess the long-term effects of this intervention.

In conclusion, this study demonstrates that multidisciplinary collaborative jejunal nutrition care significantly improves the nutritional status, pain relief, wound healing, and quality of life of burn patients. This model, through the integration of expert care, psychological support, and advanced nutritional interventions, shows great potential in improving the rehabilitation outcomes of burn patients. However, future research needs to further explore the scalability, cost-effectiveness, and long-term impact of this intervention to facilitate its broader application in clinical practice. Despite its resource-intensive nature, this integrated care model provides valuable clinical reference for burn management and deserves further research and promotion.

## Author contributions

**Conceptualization:** Fang Zou, Dan Sun, Jiang Chang, Chonggen Huang, Lingtao Ding.

**Data curation:** Fang Zou, Dan Sun, Jiang Chang, Lingtao Ding.

**Formal analysis:** Fang Zou, Dan Sun, Jiang Chang, Lingtao Ding.

**Investigation:** Fang Zou, Dan Sun, Jiang Chang, Chonggen Huang, Qing Zhou, Lingtao Ding.

**Methodology:** Fang Zou, Dan Sun, Jiang Chang, Chonggen Huang, Lingtao Ding.

**Supervision:** Dan Sun.

**Validation:** Fang Zou, Chonggen Huang, Qing Zhou.

**Visualization:** Qing Zhou

**Writing – original draft:** Fang Zou, Dan Sun, Lingtao Ding.

**Writing – review & editing:** Fang Zou, Dan Sun, Lingtao Ding.
